# Neuron-Specific Deletion of Peroxisome Proliferator-Activated Receptor Delta (PPARδ) in Mice Leads to Increased Susceptibility to Diet-Induced Obesity

**DOI:** 10.1371/journal.pone.0042981

**Published:** 2012-08-20

**Authors:** Heidi E. Kocalis, Maxine K. Turney, Richard L. Printz, Gloria N. Laryea, Louis J. Muglia, Sean S. Davies, Gregg D. Stanwood, Owen P. McGuinness, Kevin D. Niswender

**Affiliations:** 1 Department of Molecular Physiology and Biophysics, Vanderbilt University School of Medicine, Nashville, Tennessee, United States of America; 2 Department of Veterans Affairs, Tennessee Valley Healthcare System, Nashville, Tennessee, United States of America; 3 Department of Medicine, Division of Diabetes, Endocrinology and Metabolism, Vanderbilt University School of Medicine, Nashville, Tennessee, United States of America; 4 Neuroscience Graduate Program, Vanderbilt University, Nashville, Tennessee, United States of America; 5 Department of Pharmacology, Vanderbilt University School of Medicine, Nashville, Tennessee, United States of America; 6 Department of Pediatrics, Vanderbilt University School of Medicine, Nashville, Tennessee, United States of America; University of Bari & Consorzio Mario Negri Sud, Italy

## Abstract

Central nervous system (CNS) lipid accumulation, inflammation and resistance to adipo-regulatory hormones, such as insulin and leptin, are implicated in the pathogenesis of diet-induced obesity (DIO). Peroxisome proliferator-activated receptors (PPAR α, δ, γ) are nuclear transcription factors that act as environmental fatty acid sensors and regulate genes involved in lipid metabolism and inflammation in response to dietary and endogenous fatty acid ligands. All three PPAR isoforms are expressed in the CNS at different levels. Recent evidence suggests that activation of CNS PPARα and/or PPARγ may contribute to weight gain and obesity. PPARδ is the most abundant isoform in the CNS and is enriched in the hypothalamus, a region of the brain involved in energy homeostasis regulation. Because in peripheral tissues, expression of PPARδ increases lipid oxidative genes and opposes inflammation, we hypothesized that CNS PPARδ protects against the development of DIO. Indeed, genetic neuronal deletion using Nes-Cre loxP technology led to elevated fat mass and decreased lean mass on low-fat diet (LFD), accompanied by leptin resistance and hypothalamic inflammation. Impaired regulation of neuropeptide expression, as well as uncoupling protein 2, and abnormal responses to a metabolic challenge, such as fasting, also occur in the absence of neuronal PPARδ. Consistent with our hypothesis, KO mice gain significantly more fat mass on a high-fat diet (HFD), yet are surprisingly resistant to diet-induced elevations in CNS inflammation and lipid accumulation. We detected evidence of upregulation of PPARγ and target genes of both PPARα and PPARγ, as well as genes of fatty acid oxidation. Thus, our data reveal a previously underappreciated role for neuronal PPARδ in the regulation of body composition, feeding responses, and in the regulation of hypothalamic gene expression.

## Introduction

Obesity is a serious health problem in the United States and worldwide [1], [2]. Evidence indicates that body weight and adiposity can be tightly physiologically regulated through the coordinated action of distributed neurons and brain circuits, which regulate feeding and energy expenditure in response to changes in circulating hormones [3] and nutrients [4]. Dietary fat consumption, in particular, is associated with weight gain, obesity and metabolic disease [5], [6], [7], [8]. Consumption of a high-fat diet (HFD) has been shown to lead to lipid accumulation and inflammatory signaling in key neuronal subsets involved in the regulation of energy homeostasis [9], [10], [11], [12], resulting in behavioral and biochemical resistance to insulin, leptin and other regulatory hormones and nutrient signals in the CNS.

In order to understand the effects of dietary fat on obesity predisposition, we sought to identify molecular metabolic regulators that may be lipid sensitive. Peroxisome proliferator-activated receptor δ (PPARδ) is a member of the PPAR family of nuclear receptors, a class of lipid activated transcription factors belonging to the nuclear receptor superfamily [13], [14]. The three known PPAR isoforms, PPARα, PPARγ and PPARδ, display isotype-specific target gene [15], ligand binding [16] and tissue distribution patterns [17]. PPARγ regulates adipogenesis and is the target of the thiazolidinedione (TZD) class of insulin sensitizing drugs [18], while PPARα regulates genes involved in hepatic fatty acid oxidation (FAO) [19] and lipoprotein metabolism [20] and is the molecular target of the fibrate class of dyslipidemia drugs [21]. PPARδ is ubiquitously expressed and plays key roles in lipid metabolism, muscle fiber type composition and skin health [22], [23], [24]. Several chemical PPARδ agonists exist [25], but none are currently approved for use in humans. PPARs also have potent anti-inflammatory effects through transcriptional regulation of pro-inflammatory gene expression, both in the periphery [26] and central nervous system (CNS) [27].

All three PPAR isoforms are expressed to different degrees in the CNS [28]. Recent evidence suggests that CNS activation of PPARα and/or PPARγ may contribute to weight gain and obesity. Deletion of PPARγ in neurons [29] or chemical inhibition of PPARγ in the hypothalamus protects against the development of diet-induced obesity (DIO) [30]. Activation of this receptor with HF feeding or a chemical agonist increases weight gain [30], raising the possibility, at least, that HFD consumption activates neuronal PPARγ as a pathogenic mechanism in obesity. Activation of hypothalamic PPARα was also shown to correct the hypophagic phenotype in a model of increased CNS fatty acid sensing [31]. Although limited, this evidence supports a key role for PPARs in central energy homeostasis regulation.

PPARδ is the most highly expressed isoform throughout the CNS and is enriched in areas known to be involved in energy homeostasis, such as mediobasal hypothalamus [28], [32], [33]. Accumulating evidence supports a role for CNS PPARδ activation in preventing oxidative stress and inflammation in several neurodegeneration models [34], [35]. Evidence from various rodent models suggests that hypothalamic lipid accumulation and low-grade inflammation are associated with obesity [9], [12]. Given the known role of PPARδ in the regulation of genes that promote lipid oxidation [22] and its recognized anti-inflammatory effects in the CNS [36], we hypothesized that loss of PPARδ function, via genetic deletion, would lead to or potentiate obesity.

We generated neuronal PPARδ knockout mice (KO) using Nes-Cre loxP technology [37]. Cre-mediated recombination leads to deletion of exon 4, which encodes the DNA binding domain of PPARδ. On a chow diet, KO mice have increased fat mass, despite reduced body weight and lean mass. Elevated hypothalamic inflammation is accompanied by leptin resistance as well as abnormal feeding and neuroendocrine responses to fasting. Consistent with our hypothesis, KO mice are extremely susceptible to DIO, yet are surprisingly resistant to HF diet-induced elevations in CNS inflammation and lipid accumulation. Gene expression analysis revealed increased expression of genes of fatty acid oxidation and of the other PPARs with HF feeding, which may account for the lack of further increase in inflammation and lipotoxicity.

## Results

### Neuronal PPARδ knockdown and brain morphology

**Figure 1:**
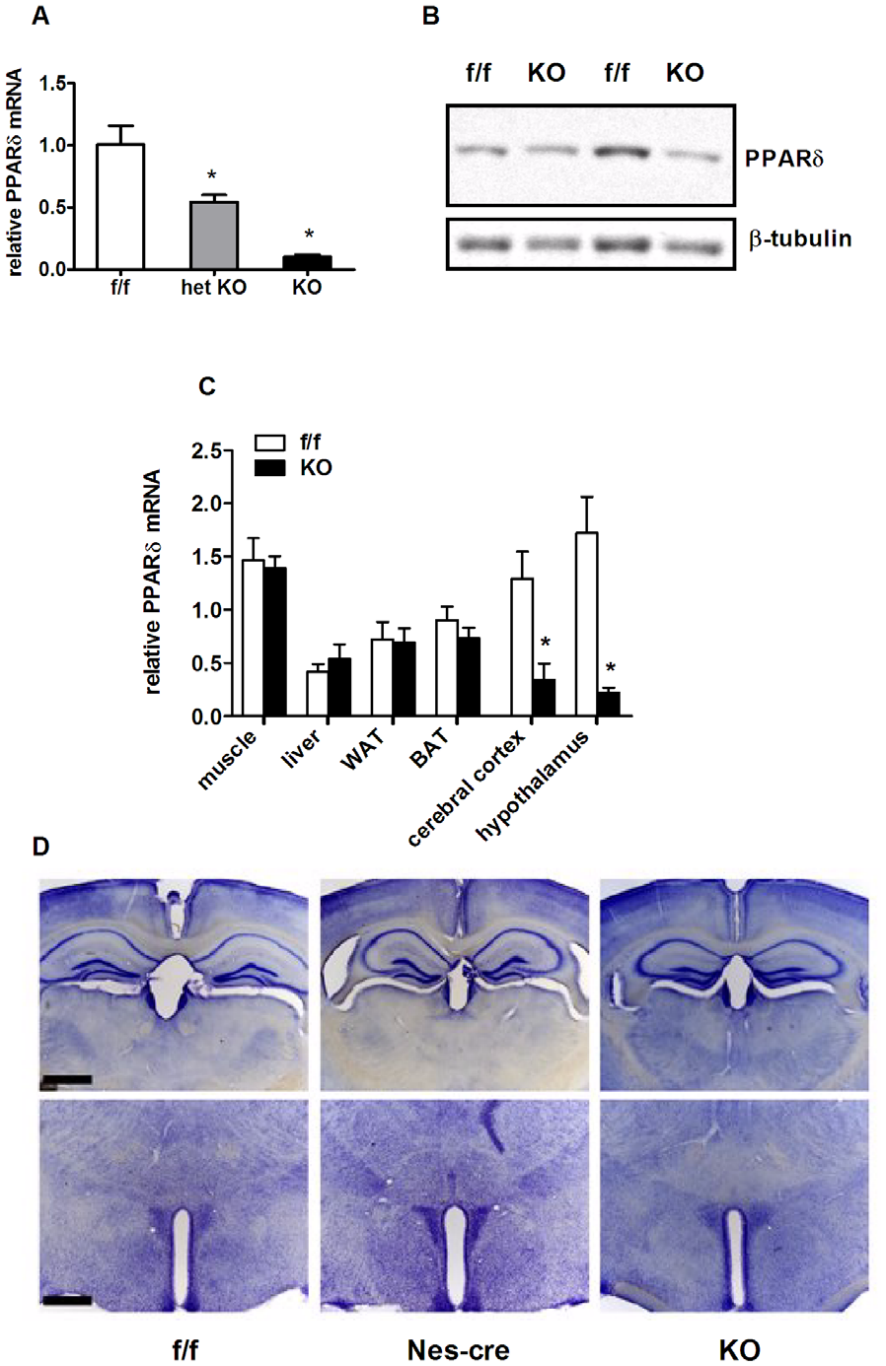
Neuronal PPARδ knockdown and brain morphology. (A) PPARδ gene expression in mediobasal hypothalamus of control (f/f), heterozygous KO (het) and homozygous KO (KO) PPARδ mice. Target gene PPARδ mRNA expression was measured by RT PCR and normalized to endogenous levels of the housekeeping gene RPL13A. (B) Representative Western blot of PPARδ protein levels in total cellular protein extracts from mediobasal hypothalamus of f/f and KO mice. β-tubulin was used as a loading control. (C) Quantification of PPARδ mRNA expression in peripheral and CNS tissues of f/f and KO mice (muscle, liver, white adipose tissue (WAT), brown adipose tissue (BAT), cerebral cortex and hypothalamus). Gene expression was measured by RT PCR and normalized to endogenous levels of the housekeeping gene RPL13A (n = 4–8). (D) Photomicrographs of Nissl staining in brains from f/f, nestin cre+ control and KO mice. Representative sections shown at the level of the hippocampus (top) and hypothalamus (bottom). No obvious differences or malformations in the structure of these or any other forebrain nuclei were observed across genotypes. Scale bar = 500 µm. Values in panels A and C represent the genotype group mean ± SEM, expressed relative to the levels of the f/f control group. Statistical significance is designated as * (*p*<0.05, *vs.* f/f control group, ANOVA or two-tailed student's *t* test).

In order to generate a neuronal loss-of-function PPARδ allele, we crossed mice with a floxed PPARδ allele [38] with mice expressing Cre recombinase under control of the rat nestin promoter [39]. Cre mediated recombination leads to excision of exon 4, which encodes the DNA binding domain of PPARδ. Double heterozygous mice were crossed to Cre negative, homozygous floxed females to produce study animals. Heterozygous (Het) and homozygous neuronal PPARδ knockout (KO) mice were born at the expected Mendelian ratios (not shown), were fertile and had no apparent developmental abnormalities compared to floxed littermate (f/f) control mice (not shown). PPARδ mRNA expression in hypothalamus was reduced in a gene dosage dependent manner (Fig. 1A), but was not altered in peripheral tissues (muscle, liver, white and brown adipose; Fig. 1C). Western analysis revealed reduction of PPARδ protein in mediobasal hypothalamus (Fig. 1B), although Western analysis for PPARs has proven technically difficult.

Because PPARδ has been noted to have roles in brain development [24], we determined whether the CNS is grossly altered by deletion of the delta isoform. Nissl stain (Fig. 1D) of coronal sections at the level of the hippocampus (top panel) and hypothalamus (bottom panel) revealed no obvious differences or malformations in the structure of these or any other forebrain nuclei between KO mice and f/f “floxed” or nestin expressing unfloxed negative controls, indicating that deletion of PPARδ in neurons does not cause major structural defects. Therefore, we proceeded to use this model to study the effects of loss of neuronal PPARδ function in energy homeostasis.

### Neuronal PPARδ deletion leads to altered body composition and leptin insensitivity

Body weight (BW) of 5-week old, chow-fed KO mice (n = 18–22) was reduced by 13% (18.96±0.33 *vs.*16.85±0.25 g, *p<*0.001, *t* test), a difference that was largely due to a reduction in lean body mass (13.17±0.35 *vs.* 11.16±0.20 g, *p<*0.001, *t* test). Lower body weight and slightly but significantly higher fat mass (1.47±0.07 *vs.* 1.90±0.05 g, *p<*0.001, *t* test) in these animals resulted in a significant elevation in adiposity (fat mass/BW ×100) (8.38±0.48 *vs.* 12.22±0.32%, *p<*0.001, *t* test).

**Figure 2:**
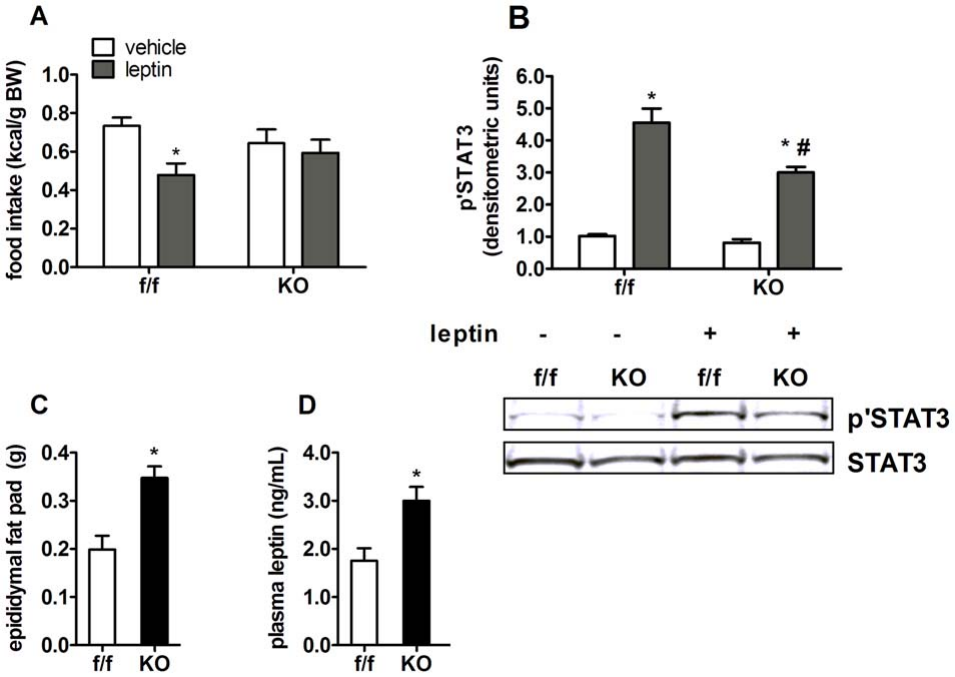
Neuronal PPARδ deletion leads to leptin insensitivity. (**A**) Food intake in chow fed f/f and KO mice after receiving a bolus injection of leptin (5 mg/kg BW, i.p.) or vehicle (saline) at the onset of the dark period. Mice were housed individually and food intake was measured over 24 hours (n = 10–12). (B) Hypothalamic total protein extracts from f/f and KO mice treated with leptin (5 mg/kg BW i.p.) or vehicle for 30 minutes were used for Western blot analysis to detect levels of STAT3 phosphorylation (Y705). Total STAT3 levels were determined and used as a loading control. Densitometery of blots yielded relative intensity of protein levels, which are expressed as an activation index (pSTAT3/total STAT3) and represented as the group mean ±SEM (n = 4–6) relative to the f/f saline group. (C) Epigonadal fat pad mass and (D) plasma leptin levels of aged matched, chow fed f/f and KO mice represent basal phenotype of these mice. Values represent the mean ± SEM. Statistical significance is denoted in A and B as * (*p*<0.05 leptin (gray bars) *vs.* vehicle (white bars) treated groups within each mouse genotype) or # (*p*<0.05, KO *vs.* f/f mice treated with leptin), and in panels C and D as * (*p*<0.05 *vs.* f/.f controls, two-tailed student's *t* test).

The adipocyte hormone leptin acts as an adiposity negative feedback signal, controlling fat mass [3], [40] through the coordinated regulation of food intake and energy expenditure. Resistance to the behavioral and biochemical effects of leptin is a hallmark of obesity [41]. Leptin treatment (5 mg/kg BW, i.p.) reduced 24 hour caloric intake, (kcal/g BW) by 24% in f/f mice (Fig. 2A) compared to vehicle, but failed to reduce food intake in KO mice (Fig. 2A). STAT3 is a direct, molecular target of leptin receptor activation [42] and its phosphorylation state can be used as a biochemical marker of leptin sensitivity. Leptin treatment (5 mg/kg BW, i.p.) significantly increased phosphorylation of STAT3 (Y705; Fig. 2B) in hypothalami of f/f mice, but this effect was significantly blunted in KO mice (Fig. 2B). These findings occurred in the context of a near doubling of epididymal adipose tissue (Fig. 2C) and higher circulating basal leptin levels (Fig. 2D), altogether suggesting that deletion of PPARδ blunts leptin sensitivity in mediobasal hypothalamus.

### Increased susceptibility to diet-induced obesity in KO mice

Based upon evidence that PPARδ is activated by dietary fatty acids [43], we hypothesized that PPARδ may be an important molecular determinant of susceptibility to environmentally induced obesity. To test this, we placed mice on a diet with high-fat (HF) content (45% kcal as fat, HFD) or a micronutrient match control diet with low-fat (LF) content (10% kcal as fat, LFD) at 5 weeks of age.

**Figure 3:**
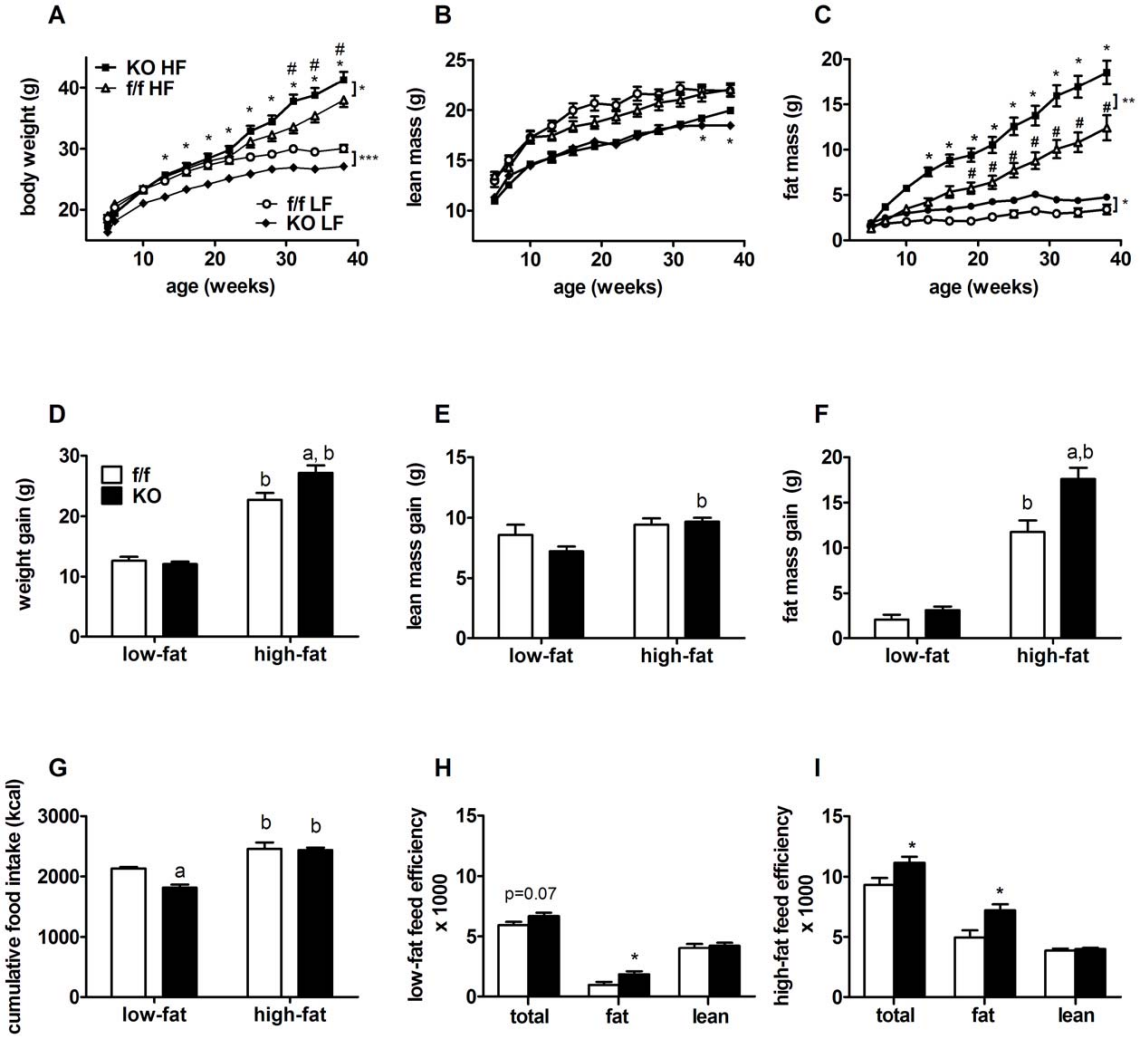
Neuronal PPARδ deletion leads to increased susceptibility to diet induced obesity. Growth curves of f/f and KO mice fed LFD or HFD for 33 weeks (age 5–38 weeks). Body weight (BW) and body composition (fat and lean mass) were measured at the indicated ages by MRI/NMR. (A) BW curves, (B) lean mass curves, (C) fat mass curves. Susceptibility to diet-induced obesity was measured by two-way repeated measures ANOVA over the experimental period (*, *p*<0.05, f/f mice fed LF *vs.* HF diet) and (#, *p*<0.05, KO mice fed LF *vs.* HF diet). Total weight gain over the experimental period for (D) body weight gain, (E) lean mass gain, (F) fat mass gain are shown for f/f and KO mice. (G) Cumulative food intake (kcal) of f/f and KO groups fed either LF or HF diets for 33 weeks, determined from bi-weekly measurements of food intake in genotype matched, group housed mice. Feed efficiency of f/f and KO mice fed (H) LFD and (I) HFD is the ratio of weight gained (BW, fat and lean) to that of calories consumed over the entire study period. Values represent the mean±SEM. Statistical significance is denoted by ^a^ (*p*<0.05, f/f *vs.* KO, same diet) or *^b^* (*p*<0.05, LF *vs.* HF, same genotype), as determined by one-way ANOVA and Bonferroni post test, or by * (*p*<0.05 *vs.* f/f controls), when determined by two-tailed student's *t* test.

Although smaller at weaning, KO mice have normal growth and gain a similar amount of weight as f/f mice over 33 weeks of LFD feeding (Fig. 3A, D). On HFD, KO mice rapidly gain weight and become significantly heavier than KO mice fed LFD after 8 weeks and surpass the body weight of HFD fed f/f controls after 21 weeks on HFD (Fig. 3A). Ultimately KO mice gained 16% (∼5 g, Fig. 3D) more body weight and were 6% heavier than f/f mice fed the same diet (Fig. 3A, Table 1), revealing a role for neuronal PPARδ expression in the determination of body weight gain during HF feeding.

**Table 1:** Plasma hormones and metabolites after 33 weeks of low-fat (LFD) or high-fat (HFD) diet.

	LFD (10% kcal fat)		HFD (45% kcal fat)	
	f/f	KO	f/f	KO
body weight (g)	29.94±0.58	27.11±0.38^a^	39.56±0.72^b^	42.07±1.20^a,b^
fat mass (g)	3.40±0.53	4.76±0.38^a^	13.27±1.18^b^	18.55±1.29^a,b^
lean mass (g)	21.90±0.59	18.47±0.28^a^	21.16±0.34	19.95±0.30
insulin (ng/mL)	0.61±0.08	1.00±0.15	1.26±0.26	2.21±0.20^a,b^
leptin (ng/mL)	5.7±1.0	18.1±2.3^a^	64.7±21.4^b^	129.3±11.4^a,b^
FFA (mM)	0.66±0.08	0.63±0.06	0.82±0.08	0.73±0.03
TG (mg/dL)	38.1±3.0	53.09±5.2^a^	63.6±5.5^b^	46.5±4.2

Metabolic characteristics, plasma hormones and metabolites were measured after 33 weeks of HFD or LFD feeding. Body composition was measured by NMR. Insulin and leptin levels were measured by RIA. FFA and TG were quantified as described in methods and materials. Mice were fasted for 4 hours prior to plasma collection. Values represent group mean mean±SEM (n = 9–11) and statistical significance is designated by a (p<0.05, f/f vs. KO, same diet) or b (p<0.05, LFD vs. HFD, same genotype), as determined by two-way ANOVA followed by Bonferroni post test.

Differences in body composition can have profound effects on the metabolic implications of weight gain. On LFD, KO mice have lower lean mass (Fig. 3B, Table 1) and slightly reduced lean mass gain (Fig. 3E), whereas HFD feeding lead to identical lean mass gain (Table 1).

At baseline, KO mice have slightly more fat mass (Fig. 3C, Table 1), and while on LFD, maintain a similar degree of elevated fat mass over time (Fig. 3F). Consumption of HFD induces obesity in both groups, but the degree of DIO is augmented in the KO group. KO animals have 6 grams, or 33% more fat mass (Fig. 3C, F, Table 1), than HFD fed f/f mice. Thus, increased body weight gain in KO mice on HFD is due to a profound accumulation of fat mass. These data reveal a significant interaction between CNS PPARδ and dietary fat exposure in DIO.

### Impact of Neuronal PPARδ deletion on food intake and energy expenditure

Impaired neuroendocrine regulation of energy balance leads to obesity. Absolute food intake was reduced in KO mice on LFD, whereas no differences in cumulative food intake were observed on HFD (Fig. 3G). Feed efficiency (calculated as the number of consumed calories required to gain 1 gram of mass) was elevated for body weight gain on HFD, and for fat mass gain on both LFD and HFD, in KO mice relative to controls (Fig. 3H, I). Interestingly, feed efficiency for lean mass gain was not different in control mice on either diet (Fig. 3H, I). Together these data suggest that KO mice are more efficient at storing calories as fat.

To determine if reduced energy expenditure contributes to increased fat mass gain in KO mice, we measured energy expenditure (EE) by indirect calorimetry. Interestingly, after 20 weeks of HFD exposure, daily EE (kcal) and EE normalized to total body weight (kcal/g BW) were not different in KO mice relative to f/f mice (Table 2). At this time point, KO animals had similar total body weight (Fig. 1A, Table 2) but reduced lean mass (Fig. 1B, Table 2) relative to controls. When normalized per gram lean mass (kcal/gran lean body mass), KO mice exhibit a slight but significant elevation in EE over 24 hours, relative to f/f mice (Table 2). During the measurement period, both total food intake (kcal/day) and food intake normalized to body weight (kcal/day/gram BW) in KO mice were similar to that of f/f controls. When food intake was normalized to lean mass (kcal/day/gram lean mass), KO mice exhibited increased intake (Table 2).

**Table 2:** Energy homeostasis analysis after 20 weeks of high-fat diet (HFD) exposure.

	f/f	KO
BW (g)	30.10±1.44	30.90±1.61
lean mass (g)	19.05±0.37	16.95±0.42**
Daily EE (kcal)	11.11±0.18	11.01±0.27
Daily EE (kcal/g BW)	0.38±0.01	0.35±0.01
Daily EE (kcal/g lean mass)	0.58±0.01	0.65±0.01**
RER light period	0.91±0.01	0.86±0.01
RER dark period	0.86±0.01	0.82±0.02
Daily FI (kcal)	10.78±0.24	10.43±0.62
Daily FI (kcal/g BW)	0.37±0.01	0.37±0.01
Daily FI (kcal/g lean mass)	0.58±0.02	0.64±0.02**

Energy expenditure (EE) and respiratory exchange ratio (RER) were measured over 24 hours by indirect calorimetry in individually housed f/f and KO mice after 20 weeks on HFD (n = 4). Values for EE (kcal/hour) and food intake were also normalized to body weight and lean body mass measured by NMR. Mean ± SEM,

* p<0.05,

** p<0.01,

*** p<0.001 KO vs. f/f same diet, Student's *t* test.

Consistent with elevated adiposity, KO mice had significantly higher plasma leptin levels on chow diet (Fig. 2D) and on both LFD and HFD (Table 1). Insulin was also elevated in KO mice (Table 1), but only on HFD, and was accompanied by modest effects on glucose tolerance (area under the glucose curve; AUC, Fig. S1A, B) on both LFD and HFD. The stress hormone corticosterone is associated with elevated adiposity and insulin resistance; nadir, peak and stress induced plasma corticosterone levels were not altered in KO mice (Fig. S1C), ruling out gross abnormalities in the hypothalamic-pituitary-adrenal axis as a cause for elevated fat mass gain.

### Effects of dietary fat and PPARδ deletion on brain lipids, fatty acid composition, and lipid metabolism genes

**Figure 4:**
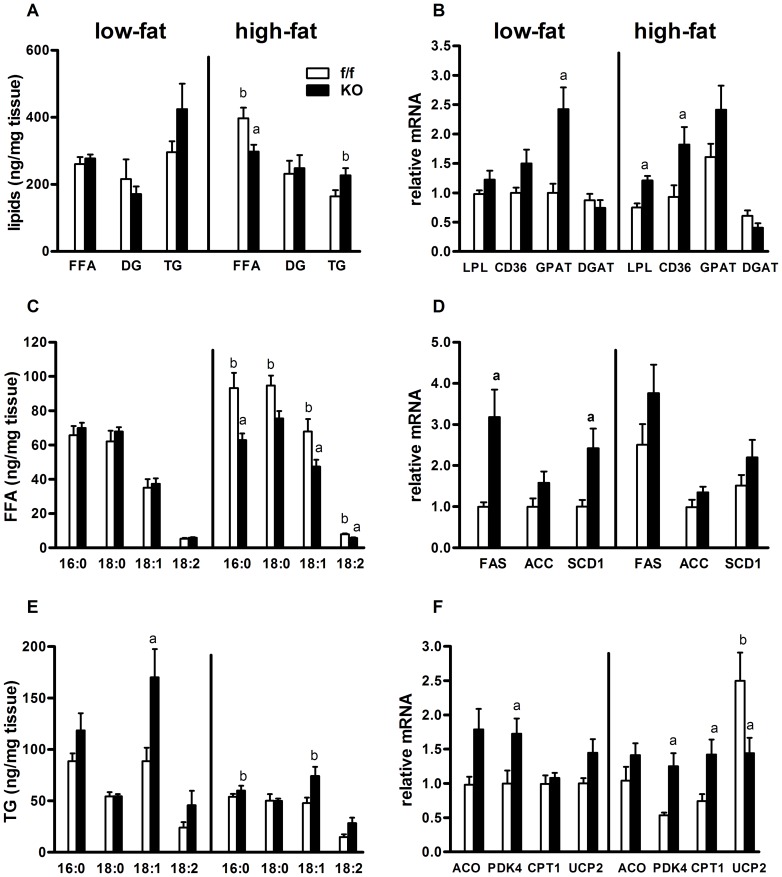
Effects of dietary fat and PPARδ deletion on brain lipids, fatty acid composition and lipid metabolism genes. (A) Total levels of triglyceride (TG), diglyceride (DG) and free fatty acid (FFA) extracted from total lipids from brains of f/f and KO mice fed LFD or HFD for 33 weeks (n = 6–7). Composition of individual FFA species making up the (C) FFA and (E) TG fractions were determined by GC-MS analysis, normalized to brain tissue mass (ng/mg tissue) and shown as group mean±SEM. (B) Changes in hypothalamic mRNA levels of target genes involved in (B) lipid uptake and storage (LPL, CD36, GPAT and DGAT), (D) lipid synthesis (FAS, ACC, SCD) and (F) fatty acid oxidation (ACO, PDK4, CPT1A, UCP2) were assessed by quantitative real-time PCR. Gene expression levels were normalized to endogenous RPL13A levels and are expressed as group mean±SEM relative to the level of the f/f LF diet control group. Statistical significance is designated as ^a^ (*p*<0.05, f/f *vs.* KO, same diet) or *^b^* (*p*<0.05, LF *vs.* HF, same genotype), as determined by one-way ANOVA and Bonferroni post test.

Hypothalamic lipid accumulation/lipotoxicity is implicated in obesity [9]. On a LFD, loss of PPARδ did not alter total brain lipid (free fatty acid (FFA), diglyceride (DG), triglyceride (TG)) content (Fig. 4A). On HFD, in control animals, total brain FFA levels were increased by 1.5-fold, while conversely, KO animals displayed no change in brain FFA levels relative to LFD (Fig. 4A) and were lower than controls on HFD. In order to identify a potential transcriptional mechanism, we assessed the expression level of genes involved in lipid uptake (lipoprotein lipase (LPL) and cluster of differentiation 36 (CD36)) and triglyceride storage (glycerol-3-phosphate acyltransferase (GPAT) and diacylglycerol acyltransferase (DGAT)). On LFD, gene expression of LPL, CD36 and DGAT were similar between f/f and KO groups, while GPAT expression was increased by 2.4-fold in KO mice on LFD relative to LFD controls (Fig. 4B). KO mice expressed higher levels of LPL and CD36 on HFD, compared to HFD fed f/f control mice (Fig. 4B), while DGAT expression did not change (Fig. 4B). GPAT expression did not change in HFD fed KO mice, relative to LFD fed, however on HFD, was no longer significantly elevated above controls (Fig. 4B).

Levels of individual FFA species were similar between genotypes on LFD (Fig. 4C). In f/f control animals, HFD increased the prevalence of saturated FFAs, palmitate (16∶0) by 1.5-fold and stearate (18∶0) by 1.4-fold, and the monounsaturated FFA oleate (18∶1) by 1.2-fold (Fig. 4C). PPARδ deletion prevented a futher accumulation of these common dietary FFAs in the CNS on HFD, relative to LFD (Fig. 4C). To determine if lipogenesis contributed to elevations in FFA levels in f/f mice, gene expression of the key lipogenic enzymes, fatty acid synthase (FAS) and acetyl-CoA carboxylase (ACC), as well as stearoyl CoA desaturase 1 (SCD1) were determined. On LFD, FAS expression was 3.1-fold higher in KO mice compared to f/f mice. HFD increased FAS in f/f mice (Fig. 4D), but did not alter expression in KO mice. Expression of SCD1 was also elevated by 2.4-fold in KO mice on LFD, compared to f/f mice. On HFD, SCD1 expression was not different between the groups. Gene expression of ACC was similar between the groups (Fig. 4D).

In agreement with increased expression of FAS, GPAT and SCD1, KO mice had elevated triglyceride levels composed of 18∶1 on LFD (Fig. 4E). Transition to a HFD resulted in significantly reduced levels of TG composed of 16∶0 in KO animals, while HFD led to reduced 18∶1 in TG of f/f mice but did not alter other TG lipid species (Fig. 4E). We next assessed gene expression of key genes involved lipid catabolism to determine if increased fatty acid oxidation might contribute to decreased lipid accumulation in brains of KO mice on HFD. On LFD, pyruvate dehydrogenase kinase 4 (PDK4) expression was increased by 1.7-fold in KO mice (Fig. 4F). HFD led to increased expression of the mitochondrial uncoupling gene uncoupling protein 2 (UCP2) in f/f mice by 2.4-fold, but did not alter UCP2 expression in KO mice (Fig. 4F). On HFD, KO mice had higher gene expression (2-fold) of two markers of mitochondrial fatty acid oxidation, carnitine palmitoyltransferase 1A (CPT1A) and PDK4 compared to f/f mice on HFD. Gene expression of acyl-CoA oxidase (ACO), a marker of peroxisomal fatty acid beta-oxidation, was not different among the groups on either diet (Fig. 4F). As a whole, these data suggest that PPAR delta could play important regulatory roles in basal CNS lipid homeostasis and responses to dietary lipid exposure.

### Gene-diet interactions determine hypothalamic inflammatory signaling and gene expression in neuronal PPARδ KO mice

**Figure 5:**
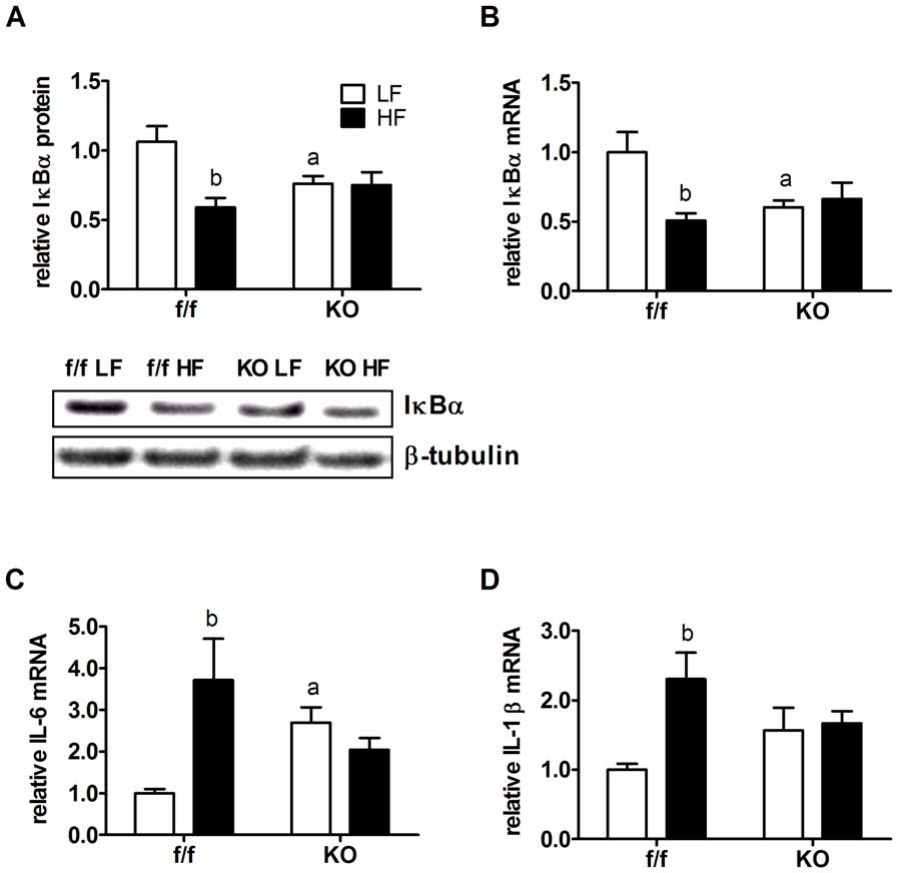
Gene-diet interactions determine hypothalamic inflammatory signaling and gene expression in neuronal PPARδ KO mice. Protein and mRNA were isolated from bisected sections of mediobasal hypothalamus of f/f and KO mice fed LFD or HFD for 33 weeks. (A) Total hypothalamic protein extracts were subjected to Western blot analysis using an antibody directed against IκBα. Levels of β-tubulin were determined and used as a loading control. Densitometery of blots yielded relative intensity of protein levels (n = 6). Insert of A shows a representative Western blot. Hypothalamic mRNA levels of (B) IκBα and inflammatory cytokines (C) IL-6 and (D) IL-1β in f/f and KO mice measured by RT-PCR after the study period. Target gene mRNA levels were normalized to endogenous RPL13A levels. Values are represented as group mean ± SEM relative to the LF f/f control group. Statistical significance is designated by ^a^ (*p*<0.05, f/f *vs.* KO, same diet) or *^b^* (*p*<0.05, LF *vs.* HF, same genotype), as determined by two-way ANOVA and Bonferroni post test.

Activation of PPARδ has anti-inflammatory effects in both the periphery and CNS [34], [44]. In order to determine if loss of PPARδ increases inflammatory signaling in hypothalamus, we measured levels of the cytoplasmic inhibitory protein of NF-κB, IκBα, and the pro-inflammatory cytokines, IL-6 and IL-1β, which are two transcriptional targets of NF-κB [45]. Interestingly, IκBα levels in hypothalamic total cell extracts were reduced by 25% in KO mice fed LFD, compared to LFD fed f/f mice (Fig. 5A). In response to dietary fat exposure, IκBα levels were reduced by ∼40% (Fig. 5A) in f/f hypothalami, as reported in several models and is consistent with lipotoxicity and activation of pro-inflammatory signaling [9], [10]. Interestingly, HFD feeding in KO animals failed to further reduce IκBα protein levels (Fig. 5A). An identical response was observed for IκBα mRNA levels (Fig. 5B). Consistent with IκBα data, KO animals have increased hypothalamic IL-6 gene expression on LFD relative to controls (Fig. 5C). While HFD increased IL-6 gene expression, and another NF-κB target pro-inflammatory gene, IL-1β, in f/f controls, KO animals were protected from further HFD induced increases in hypothalamic inflammatory cytokine gene expression (Fig. 5C, D).

### Adipose tissue hypertrophy and inflammation

**Figure 6:**
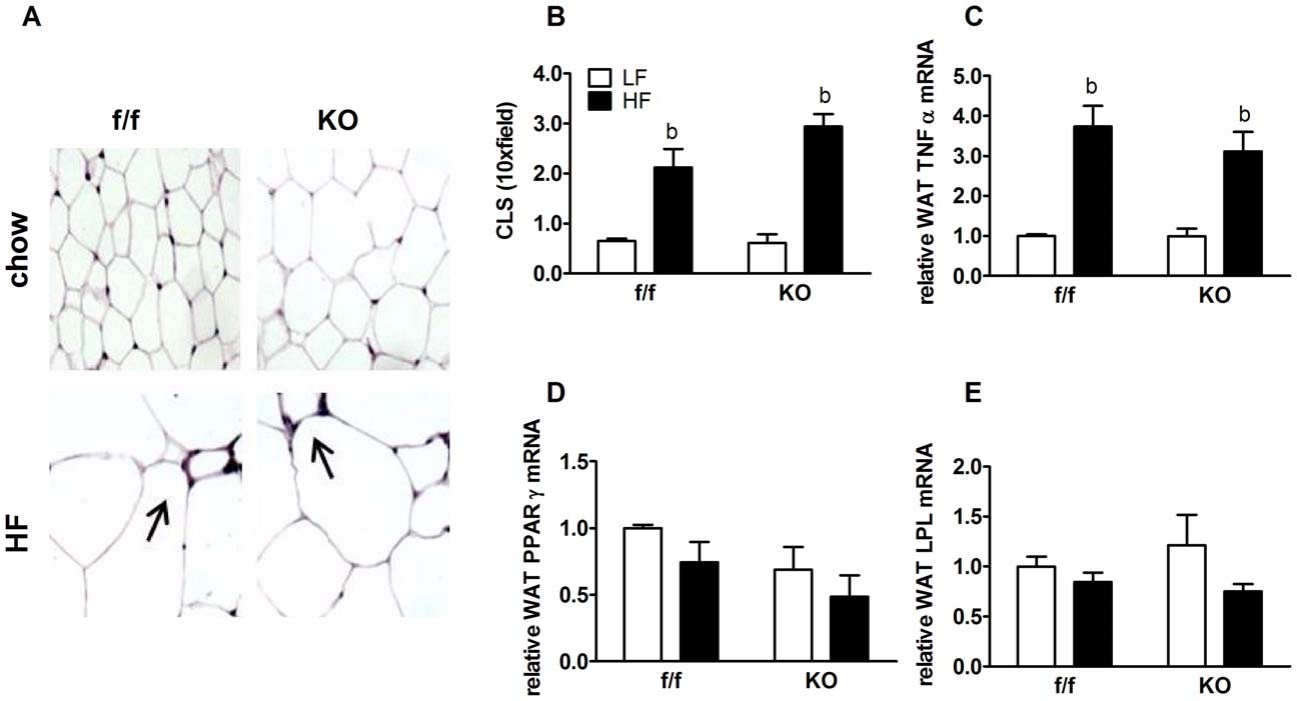
White adipose tissue hypertrophy and inflammation. (**A**) Light micrographs (×10 magnification) of H&E stained slides of WAT from f/f and KO mice fed chow or HFD for 33 weeks. Arrows point to crown-like structures (CLS), of areas of macrophage infiltration and inflammation. (B) Quantification of CLS (CLS/10× field, n = 4) corresponding to inflammatory macrophage infiltration around adipocytes in f/f and KO mice fed either a chow diet or a HFD. Adipose gene expression of inflammatory cytokine (C) TNFα and adipogenesis markers (D) PPARγ and (E) LPL measured by RT PCR. Target gene mRNA levels were normalized to endogenous RPL13A levels. Values are represented as group mean ± SEM relative to the f/f LF group. Statistical significance is designated as *^b^* (*p*<0.05, LF *vs.* HF same genotype), as determined by two-way ANOVA and Bonferroni post test.

Adipose tissue serves as a source of circulating cytokines that promote systemic inflammation in obesity. Adipose inflammation, measured by the presence of crown-like structures (CLS) corresponding to macrophage infiltration (Fig. 6A, B), and TNFα mRNA expression (Fig. 6C), were similarly elevated in epididymal white adipose tissue (WAT) from f/f and KO mice on HFD. Increased adiposity is accompanied by adipose hypertrophy in KO mice on chow diet (Fig. 6A), but there were no differences in WAT expression of the adipogenic markers PPARγ (Fig. 6D) and LPL (Fig. 6E). Collectively, these data suggest that alterations in adipocyte function or systemic inflammatory status do not explain changes (or lack thereof) in CNS inflammation in KO mice fed HFD.

### Neuronal PPARδ deletion alters regulation of hypothalamic neuropeptide gene expression and responses to prolonged fasting

**Figure 7:**
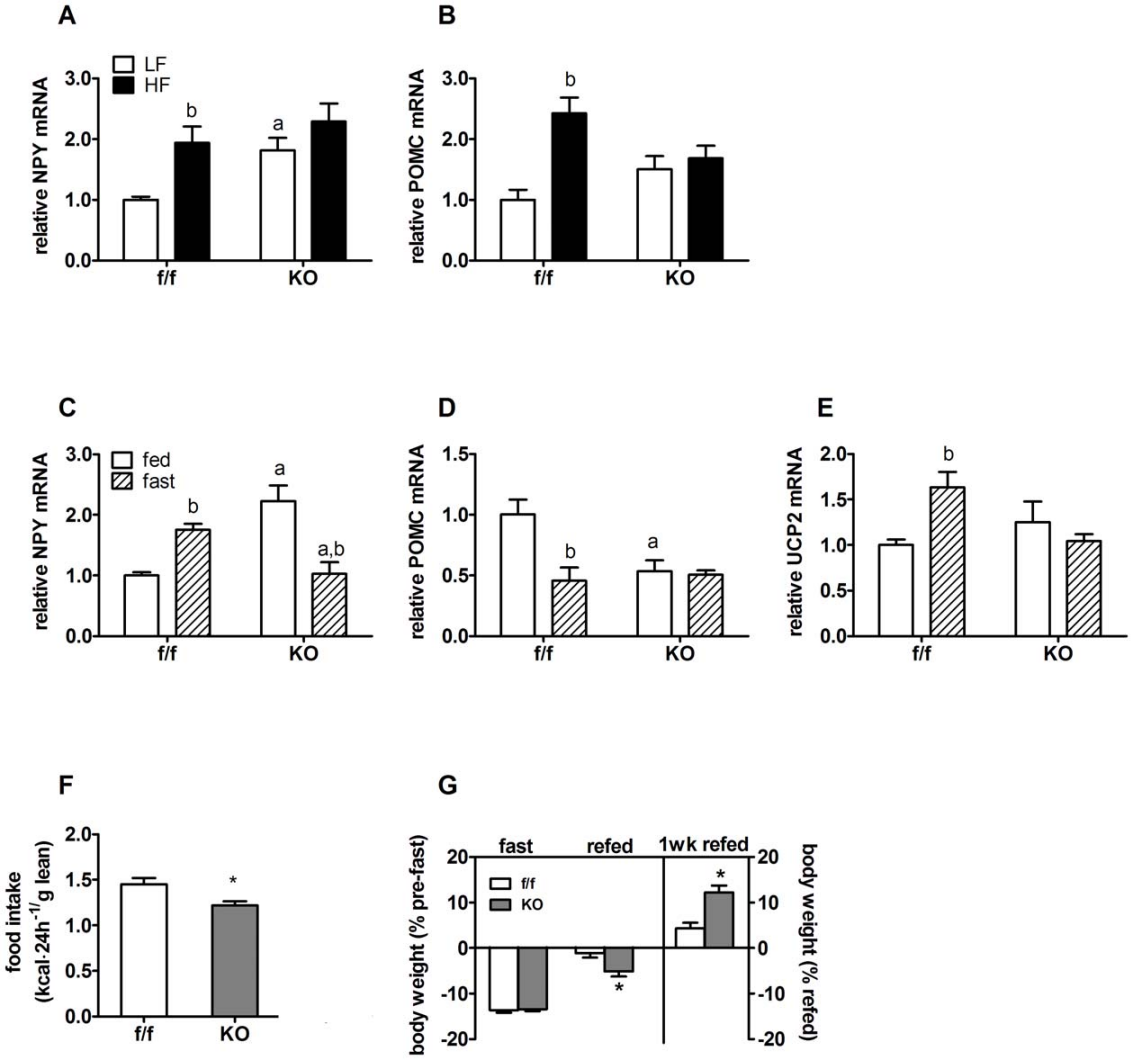
Neuronal PPARδ deletion alters hypothalamic neuropeptide gene expression and compensatory hyperphagia after prolonged fasting. Hypothalamic mRNA levels of neuropeptides in f/f and KO mice fed LFD or HFD for 33 weeks (n = 6–7). Target gene mRNA levels of (A) NPY and (B) POMC were assessed by quantitative RT PCR. (C–D) Fasting induced changes in hypothalamic neuropeptide mRNA levels of f/f and KO mice maintained on a chow diet or fasted for 24 hours. Target gene mRNA levels of (C) NPY, (D) POMC and (E) UCP2 in fed and fasted mice were normalized to RPL13A and are expressed relative to the f/f, fed control. (F) Refeeding after fasting was measured for an additional 24 hours in a separate cohort of individually housed chow fed mice (n = 6–7). Graph shows food intake normalized to basal, pre-fast lean mass (kcal/g lean mass). (G) Percent changes in body weight after a 24 hour fast, after fasting and refeeding for 24 hours, or an additional 7 days. Values represent the mean ± SEM. Statistical significance in panel A–E is designated as ^a^ (*p*<0.05 f/f *vs.* KO, same diet) or *^b^* (*p*<0.05, LF *vs.* HF, same genotype), as determined by two-way ANOVA and Bonferroni post test, and in F and G by * (*p*<0.05 *vs.* f/f), as determined by two-tailed student's *t* test.

To identify mechanisms by which loss of neuronal PPARδ increases DIO susceptibility, we measured hypothalamic mRNA expression of key regulatory neuropeptides, NPY and POMC, following HFD and LFD feeding. On LFD, NPY expression was increased by 1.8-fold in KO mic, (Fig. 7A). HFD feeding led to a 2-fold increase in NPY expression in f/f mice, but had no effect to further increase NPY in KO mice (Fig. 7A). POMC expression was not different on LFD between KO and controls (Fig. 7B). HFD increased POMC expression by 2.4-fold in f/f mice relative to LFD, but did not alter POMC in KO animals on HFD relative to LFD (Fig. 7B) despite a ∼3-fold increase in circulating leptin levels (Table 1). Collectively, these data suggest profound dysregulation of adiposity negative feedback signaling and regulation of neuropeptide gene expression in KO mice on both diets.

The orexigenic neuropeptide NPY is a powerful activator of food intake. NPY expression is potently induced by fasting and in the absence of leptin signaling [46]. In this situation, simultaneous inhibition of POMC neurons (and down regulation of anorexogenic POMC gene expression) facilitates subsequent hyperphagia and weight regain. Fasting elicited the appropriate neuropeptide expression pattern in hypothalamus of f/f mice, increasing NPY by 1.8-fold (Fig. 7C) and decreasing POMC expression by half (Fig. 7D). Fasting paradoxically decreased NPY expression in KO mice (Fig. 7C) and did not lead to reduction of POMC expression (Fig. 7D). UCP2, a known target of PPARδ regulation, has been implicated in hypothalamic nutrient sensing, and neuronal responsivity to changes in energy availability and adiposity negative feedback signaling [47]. UCP2 expression was increased 1.6-fold by fasting (Fig. 7E) in f/f mice but was unchanged in KO mice, suggesting a potential molecular mechanism for disruption of neuropeptide gene expression.

To understand the functional implications of altered neuropeptide expression in KO mice, we measured food intake in a second group of individually housed mice following a 24 hour fast. Consistent with blunted fasting induced UCP2 and NPY expression, KO mice consumed significantly fewer calories (normalized to lean mass, kcal/g lean mass) after fasting (Fig. 7F), resulting in attenuated weight regain after 24 hours of refeeding (Fig. 7G). Interestingly, KO mice gained significantly more weight than f/f mice in the 8 days following the fasting challenge (Fig. 7G), suggesting that stress-induced weight gain may be exaggerated in the longer term. Together with impaired leptin sensitivity, these data raise the possibility that loss of PPARδ function in neurons impairs both anorexogenic and orexigenic tone.

### Neuronal PPARδ deletion leads to increased hypothalamic PPARγ and PPARα expression

**Figure 8:**
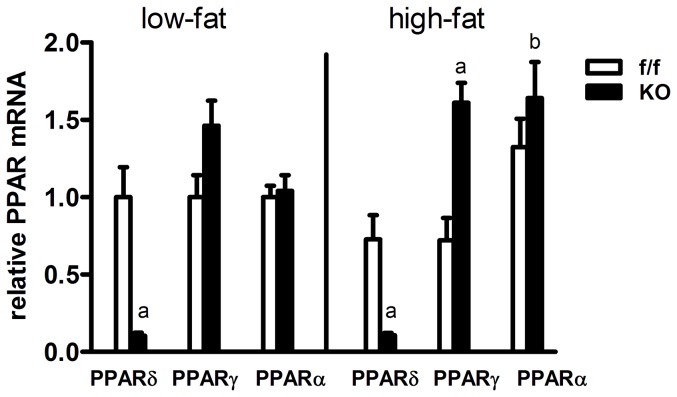
Effects of neuronal PPARδ deletion on hypothalamic PPARγ and PPARα expression. Hypothalamic mRNA expression of PPAR isoforms in f/f and KO mice fed LFD or HFD for 33 weeks was assessed by quantitative real-time PCR. Changes in PPARδ, PPARγ and PPARα were normalized to endogenous RPL13A levels and expressed relative to that of the f/f LFD group. Values represent group mean±SEM (n = 6–7). Statistical significance is designated ^a^ (*p*<0.05, f/f *vs.* KO, same diet) or *^b^* (*p*<0.05, LF *vs.* HF, same genotype), as determined by two-way ANOVA and Bonferroni post test.

We next evaluated hypothalamic expression of all the PPAR isoforms to determine if PPARα and PPARγ could be involved in variations in brain lipid content and gene expression in KO mice. Gene deletion of PPARδ resulted in a ∼90% reduction of mRNA expression, on both diets (Fig. 8A). Expression of PPARα and PPARγ were similar between f/f and KO mice fed LFD (Fig. 8). Consumption of HFD increased PPARα expression by 1.6-fold in KO mice relative to LFD fed mice, but not relative to HFD fed f/f mice (Fig. 8). Potentially consistent with reduced CNS FFA accumulation in KO mice on HFD (Fig. 4C), expression of the PPARα target gene, CPT1A, was increased, although expression levels of another target gene, ACO, was not different between genotypes (Fig. 4F). PPARγ expression was 2.6-fold higher in KO mice than f/f mice on HFD (Fig. 8), a finding that is potentially consistent with reduced hypothalamic inflammatory tone (Fig. 7) and elevated expression of the PPARγ target genes, LPL and CD36 (Fig. 4B), in KO animals on HFD.

## Discussion

Dietary fat contributes to obesity pathogenesis independent of caloric density [48]. Lipotoxicity and inflammation in key regulatory neurons and brain regions (such as mediobasal hypothalamus) are thought to contribute to positive energy balance and weight gain [9], [10], [12]. PPARδ regulates transcription of genes involved in fatty acid oxidation and has been shown to reduce inflammation and promote insulin sensitivity in peripheral tissues [22], [49], [50]. Relatively less is known about PPARδ function in the CNS where it has been implicated in neuroprotection by opposing neuronal inflammation and oxidative stress (reviewed in [36]). We sought to identify a role for neuronal PPARδ in energy homeostasis, and hypothesized that PPARδ acts to reduce lipid accumulation and inflammation, opposing the development of biochemical resistance to homeostatic signals such as leptin. Thus, neuronal deletion, we hypothesized, would lead to obesity. Consistent with this hypothesis, neuronal PPARδ deletion results in a profound susceptibility to DIO.

Interestingly, we also observed increased adiposity on LFD. The baseline phenotype is characterized by increased fat mass, lower lean mass and elevated feed efficiency on a LFD (Fig. 3, Table 1). Given the primary role of leptin in the regulation of energy balance [51], it was not surprising that KO animals exhibit blunted behavioral (Fig. 2A) and signaling responses (Fig. 2B) to leptin stimulation. These findings on LFD suggest that PPARδ plays an important role in energy homeostasis regulation, even in the absence of excess dietary fat. On a HFD, genetic loss of PPARδ function further potentiated fat mass gain (Fig. 3C), consistent with our hypothesis that PPARδ mediates protective effects against a lipotoxic environment. Interestingly, excess fat mass accrual occurred in the absence of large differences in food intake or energy expenditure (normalized to either BW or lean mass). Therefore, preferential disposition of consumed calories towards adipose tissue storage, likely coupled with subtle imbalances between food intake and energy expenditure, contributes to excess adiposity and weight gain in these animals over time. Deletion of the melanocortin-3 receptor, results in a similar fuel partitioning phenotype [52].

Given its role in the transcriptional regulation of lipid metabolism, we hypothesized that deletion would potentiate the effects of HFD on CNS lipid accumulation. Instead, neuronal PPARδ deletion did not promote lipid accumulation and opposed accumulation of FFAs in the brain of KO mice fed a HFD, despite increased expression of two genes involved in cellular lipid uptake, LPL and CD36 (Fig. 4B). The lack of rise of FFA content may be related to the relative overexpression (compared to control animals) of fatty acid oxidation genes in hypothalamus, including CPT1 and PKD4 (Fig. 4F). Brain TG content was modestly reduced in both null and control animals on HFD (Fig. 4A, E), consistent with a reduction in expression of DGAT, an enzyme required for TG synthesis. Thus, at face value, these findings in the CNS contradict the general concept that PPARδ activation opposes lipid accumulation, at least the species we measured. In reality, PPARδ utilizes several modes of transcriptional regulation and can repress basal transcription of target genes when not ligand bound, while other genes, including UCP2, do not appear to be repressed and are not upregulated by PPAR delta depletion [53], [54]. Indeed, it has been demonstrated in macrophages in vitro [55] and in cardiac tissue in vivo [56] that depletion of PPARδ has a similar effect as ligand induced activation, to increase expression of some fatty acid oxidation genes [53]. In this context, genetic deletion ultimately leads to de-repression, which is consistent with the observed upregulation of PDK4, a target gene involved in fatty acid oxidation, in our KO model (Fig. 4F).

Interestingly, despite little change or a relative reduction in brain lipids, KO animals exhibited elevated markers of hypothalamic inflammation on LFD. Surprisingly, KO animals were resistant to further activation of hypothalamic inflammation in response to HFD (Fig. 5). This was true whether we assessed IκBα, a key upstream regulator of NF-κB activity, or IL-6 and IL-1β, two key pro-inflammatory targets of NF-κB regulation. These effects could not be attributed to alterations in peripheral inflammatory mediators, as KO and control adipose tissue exhibited similar levels of markers of inflammation (Fig. 6), however, the lack of accumulation of saturated fatty acids (and presumably of lipotoxic intermediaries) may explain this. Further, PPARδ is known to interact with and sequester the nuclear corepressor and negative regulator of NF-κB, BCL-6, which is released upon ligand binding or loss of PPARδ [57].

Consistent with hypothalamic dysfunction indicated by impaired leptin responses, KO animals exhibited marked abnormalities in compensatory responses to fasting and refeeding (Fig. 7C). These findings raise the possibility of impaired stress responses, however there were no differences in baseline, nadir, or stressed levels of corticosterone (Fig. S1C). Dramatic differences in neuropeptide gene expression (both NPY and POMC) at baseline, and a complete absence of compensatory responses to fasting (Fig. 7C, D) and HFD (Fig. 7A, B), further suggest profound dysregulation of energy balance.

Interestingly, the blunted fasting induced UCP2 and NPY expression and impaired refeeding response in our neuronal KO model were similar to a global PPARδ KO model [58]. An explanation for neuropeptide dysregulation could be blunted fasting or HFD induced UCP2 expression and augmented reactive oxygen species (ROS) production. ROS serve as nutrient signals and second messengers in hypothalamic neurons, where they are known to repress NPY while simultaneously promoting POMC expression [59]. The regulatory effects of ROS to repress NPY neuronal activation and neuropeptide expression are abrogated by UCP2 mediated mitochondrial uncoupling [47]. Mice that overexpress UCP2 have elevated NPY expression but also exhibit reduced basal inflammation [60]. Conversely, mice lacking the gene for UCP2 have elevated levels in peripheral tissues of basal NF-κB activation [61] and increased cytokine expression after ischemic injury [62]. UCP2 also protects against hypothalamic injury and inflammation [63]. Although KO mice display a slight increase in basal UCP2 expression (Fig. 4F), fasting and HFD feeding failed to further increase UCP2 expression, which was associated with elevated basal inflammatory cytokine gene expression (Fig. 4 and 7).

In addressing mechanisms involved in lipid metabolism and inflammation, we observed consistent changes in the expression of several isoform specific target genes (Fig. 4) of PPARα (CPT1A) and PPARγ (LPL) [30], [64]. Indeed, PPARα expression was slightly elevated, while PPARγ was significantly elevated in brains of KO mice on HFD (Fig. 8). Two additional target genes of PPARγ, CD36 and GPAT, were also elevated in KO mice (Fig. 4). Deletion of PPARδ in cardiomyoctes, and in vitro systems, was shown to cause a similar induction of PPARα, PPARγ and their target genes involved in fatty acid oxidation [53], [56], [65]. Activation of hypothalamic PPARα and/or PPARγ has been implicated in weight gain and obesity, potentially consistent with elevated adiposity and DIO in PPARδ KO mice. Given multiple complex modes of regulation of multiple target genes, including other PPAR isoforms [54], [66], an understanding of the relevance of PPARα and PPARγ upregulation will require further study with more specific tools across a broader range of target genes.

Collectively, our data support a model where neuronal PPARδ expression is critical to the function of regulatory neurons involved in energy homeostasis. Profound dysregulation of homeostatic responses to fasting and refeeding, an experimental maneuver to amplify potential defects in the system, reveal UCP2 (whose expression is not de-repressed [53], [54]) as a potential molecular mediator of the phenotype. The inability to upregulate UCP2 in response to normal physiological stressors and after feeding raises the possibility that hypothalamic oxidative stress is a key step in obesity pathogenesis, which may be independent of lipotoxicity, at least in this model. Future studies will be required to understand the potential roles of UCP2, inflammation and compensatory changes in other PPAR isoforms in this complex phenotype. Such studies are warranted, because PPARs are targets of dietary lipids (or metabolites thereof), and are likely to shed important new light upon plausible mechanisms by which a changing dietary environment may multifactorially enhance susceptibility to obesity.

## Materials and Methods

### Animal care

Mice were housed in a temperature (22°C) and light (12 hour light/dark cycle) controlled room with free access to food and water except where indicated. All studies were approved by the Vanderbilt University Institutional Animal Care and Use Committee and were conducted in accordance with the Guide for the Care and Use of Laboratory Animals.

### Neuronal PPARδ deletion

Neuronal PPARδ knockout (KO) mice were generated by mating B6.129S4-Ppardtm1Rev/J mice (The Jackson Laboratory) with loxP sites flanking exon 4 of the PPARδ gene [38] with B6.Cg-Tg(Nes-cre)1Kln/J mice (The Jackson Laboratory) that express Cre recombinase under the control of the rat nestin promoter [67]. Both parental mouse lines were backcrossed to C57BL/6 mice for at least 8 generations prior to breeding. Genotyping for floxed PPARδ gene exon 4 and Nes-Cre alleles were performed as described [68].

### Feeding studies in mice

Mice were fed either a standard laboratory chow diet (LabDiet 5001) or purified, micronutrient matched diets with LF content (Research Diets, D01060501, kcal% = 10% fat, 20% protein, 70% carbohydrate) or HF content (Research Diets, D12451; Kcal% = 45% fat (36% saturated fat), 20% protein, 35% carbohydrate). Body composition was measured by NMR spectroscopy (Bruker Optics).

### Energy expenditure

Energy expenditure was assessed by indirect calorimetry in 12 week old chow fed mice and after 20 weeks on HFD. Mice were housed individually in Oxymax cages (Columbus Instruments; Columbus, Ohio). VO_2_ and VCO_2_ (mL/hour) were calculated based on the input and output rates of O_2_ consumption and CO_2_ production, which were used to determine the respiratory exchange ratio (RER = VCO_2/_VO_2_) and heat (kcal/hour = (3.815+1.232×RER)×(VO_2_)) using the provided software. EE data (kcal/hour) was also normalized to body weight (g) and lean mass (g) measured by NMR the day mice were placed in the chambers.

### Histology

Mice were anesthetized with sodium pentobarbital (60 mg/kg) and transcardially perfused with 4% paraformaldehyde. Brains were removed and post-fixed overnight, sucrose embedded, coronally sectioned at 40 µm and Nissl stained as previously described [69]. Epididymal white adipose tissue samples were fixed for 24 hours in 4% paraformaldahyde, transferred to 70% ethanol before paraffin embedding and staining with hematoxylin and eosin (H&E) stain. Series of nonadjacent sections were processed for each staining protocol. Images were collected at 10× magnification using bright field microscopy and qualitatively examined by an experimenter blinded to genotype.

### Medial basal hypothalamus wedge dissection

Medial basal hypothalamus dissection was performed as described [70]. Wedges were bisected along the third ventricle in some cases to allow for protein and gene expression analysis.

### Leptin sensitivity

Individually housed, male, KO and control (f/f) mice were acclimated to intraperitoneal (i.p.) saline injections (300 µl) for 7 days. Mice were given an i.p. injection of leptin (5 µg/g body weight; ProSpec, East Brunswick, NJ) or vehicle (saline) at the onset of the dark period and food intake was measured over 24 hours to assess behavioral leptin sensitivity. Leptin signaling was assessed in a second group of mice treated similarly. Hypothalami were collected 30 minutes after leptin was injected and processed for immunological detection of STAT3 phosphorylation (Tyrosine 705) by Western blot analysis.

### Glucose tolerance test

Mice were fasted for four hours prior to receiving an i.p. glucose bolus injection (1 g/kg). Glucose was measured in tail blood obtained from a small incision made at the tip of the tail with a sterile razor blade using a Freestyle handheld glucometer from Abbott Labs (Abbott Park, IL). Glucose levels were measured at various time points over 120 minutes and analyzed by area under the glucose curve from 0–120 minutes.

### Plasma hormones and metabolites

Trunk blood was collected at the conclusion of the studies, separated by centrifugation and stored at −80°C. Plasma levels of insulin and leptin were measured by radioimmunoassay (Hormone Assay & Analytical Services Core, Vanderbilt DRTC). Plasma triglycerides and free fatty acid levels were measured using kits from Waco Diagnostics (Richmond, VA). Corticosterone levels were measured as previously described [71].

### Fasting-refeeding challenge

Individually housed mice were weighed and food was withdrawn for 24 hours. Weight loss was assessed by a change in body weight at the end of the fasting period. Hyperphagia and weight gain were measured after a 24-hour refeeding period, during which time, the mice had free access to chow diet. Hypothalamic neuropeptide mRNA expression was measured in a second group of mice treated similarly.

### Western Blot analysis

Following 33 weeks of HFD or LFD feeding, mice were fasted for 4 hours prior to collecting tissue samples that were then stored at −80°C. Samples were processed for Western blot analysis, as previously described [9], and membranes were probed with primary antibodies against IκBα, phospho-Y705 STAT3, STAT3 and GAPDH (Cell Signaling; Danvers, MA) followed by HRP conjugated secondary antibodies (Promega; Madison, WI). Protein levels were detected using Western Lightning Plus-ECL Enhanced Chemiluminescence Substrate Kit (Perkin Elmer; Waltham, MA) and image intensity was quantified by densitometry using ImageJ (NIH).

### RT-PCR

Total RNA was extracted from frozen hypothalami using the RNAqueous kit (Ambion; Austin, TX). cDNA was synthesized using the High Capacity cDNA reverse transcription kit (Applied Biosystems; Carlsbad, CA). The resulting cDNA template was used to quantify mRNA expression via quantitative real-time PCR on a Bio-Rad iCycler using iQ SYBR green Supermix reagent (Bio-Rad; Hercules, CA). Real-time primers were designed using Beacon Design software (Palo Alto, CA). Primer sequences are found in Table S1. Gene expression was normalized to endogenous expression of the housekeeping gene RPL13A.

### Brain lipid analysis

Brain lipids were quantified as previously described [72] via extraction using the method of Folch [73], followed by thin layer chromatography(TLC) [74] and quantified using gas chromatographic analysis (GC) (Hormone Assay & Analytical Services Core, Vanderbilt DRTC).

### Statistical Analysis

Data are reported as mean ± SEM. Statistical analysis of differences was analyzed by two-way ANOVA followed by post hoc Bonferroni's multiple comparison test using GraphPad Prism version 5.0 for Windows (San Diego, CA). The student's *t* test for non-paired values was performed when two groups were compared with each other. A *p* value<0.05 was considered statistically significant.

## Supporting Information

Figure S1 Glucose tolerance and corticosterone response in neuronal PPARδ KO mice. (A) Glucose tolerance test (1 g/kg BW dextrose i.p.) in f/f and KO mice on chow or HFD for 20 weeks (n = 8–10). Mice were fasted for 4 hours and glucose measured in tail blood at the indicated time points. (B) Area under the glucose curve (AUC) analysis from glucose tolerance testing. (C) Plasma corticosterone levels in individually housed, chow fed, f/f and KO mice. Blood was collected at nadir (8am), peak (5pm) or 30 minutes after mild restraint stress. Values represent the mean ± SEM. Statistical significance is denoted in B as ^a^ (p<0.05 f/f vs. KO, same diet), and *^b^* (p<0.05 LF vs. HF same genotype), one-way ANOVA and Bonferroni post test.
(TIF)

Table S1 Real-time RT PCR primer list.
(DOCX)
