# Supplementary material for: Neuron-Specific Deletion of Peroxisome Proliferator-Activated Receptor Delta (PPARδ) in Mice Leads to Increased Susceptibility to Diet-Induced Obesity
Source: PLoS One. 2012 Aug 20;7(8):e42981. doi: 10.1371/journal.pone.0042981 (PMC3423438; doi:10.1371/journal.pone.0042981)
Supplement: Table S1 — Real-time RT PCR primer list. (DOCX) [file pone.0042981.s002.docx]

| **Table S1**. Real-time RT PCR Primer list | | | |  |
| --- | --- | --- | --- | --- |
| Gene Name | Primer sequences | |  |  |
|  | Sense 5’-3’ | Antisense 5’-3’ |  |  |
| *PPAR α* | TGTCGGGATGTCACACAATGC | TTCAGGTCGTGTTCACAGGTAAAG |  |  |
| *PPARδ* | GGATTTTAGAGTGGGTGTTTTTTA | CACACCCGATTCCATGTTGAG |  |  |
| *PPARγ* | TGGTTATTTTGTAGGTTGGTTT | TTGATCGCACTTTGGTATTCTTGG |  |  |
| *LPL* | GGAGAGTTATTTAAGAAAGGTGG | AGTCAGGCCAGCTGAAGTAGGAGT |  |  |
| *CPT1A* | AACACCATCCACGCCATACTG | TCCCAGAAGACGAATAGGTTTGAG |  |  |
| *UCP2* | TAAAGGTTAAGGTGTAAGGAAAATTAAG | GCAATGGTCTTGTAGGCTTCG |  |  |
| *GPAT* | GGTTAAAGAATTAAATTTTGTT | ACAGAATGTCTTTGCGTCCA | [[1](#_ENREF_1)] |  |
| *SCD-1* | GAGGGTAGTAAATTTGTTGGT | CCCAGTCGTACACGTCATTTT | [[1](#_ENREF_1)] |  |
| *ACC* | TTTGTTGGTGTTGGGATTTATAG | GTCATTACCATCTTCATTACCTCAATCTC |  |  |
| *ACO* | GGATTTTTTGTAGGAAGGAGA | CCACTCAAACAAGTTTTCATACACA | [[2](#_ENREF_2)] |  |
| *FAS* | TTAGTGATTGGAGTAGAGAGAAGTTG | ACAGAGGAGAAGGCCACAAA | [[3](#_ENREF_3)] |  |
| *DGAT2* | AGGGGTTTTTTTTAAGGTTTTTTTT | AATAGGTGGGAACCAGATCAGC |  |  |
| *PDK4* | GGGTTATAGGATGGATTGAG | CATGGAACTCCACCAAATCCA |  |  |
| *NPY* | GGATAGTTGGTAGTGTTAAAGATTT | AATCAGTGTCTCAGGGCTGGA | [[4](#_ENREF_4)] |  |
| *POMC* | GAATTTTTTTTGGGGATTAGGG | GCGAGAGGTCGAGTTTGCA | [[4](#_ENREF_4)] |  |
| *TNFα* | AGAGAGGTTGTTAGGAGTTTGGT | CTCCTCTGCTTGGTGGTTTG | [[5](#_ENREF_5)] |  |
| *IL-6* | TTTTAAAGGAGTTTTTGGAAAAG | GCATCATCGTTGTTCATAC | [[6](#_ENREF_6)] |  |
| *IKBα* | AGTTTGGTTAATAAGGGTGAGG | CAAAGTCACCAAGTGCTCCACGAT | [[5](#_ENREF_5)] |  |
| *IL-1β* | GGAAAATTTTTTTAGGAGGTGATT | GATCCACACTCTCCAGCTGCA | [[5](#_ENREF_5)] |  |
| CD36 | AATTAGTAGAACCGGGCCAC | CCAACTCCCAGGTACAATCA | [[7](#_ENREF_7)] |  |
| *RPL13A* | AGATGCACTATCCAAGA | AGTCTTTATTGGGTTCAC |  |  |
|  |  |  |  |  |

1. Biddinger SB, Hernandez-Ono A, Rask-Madsen C, Haas JT, Aleman JO, et al. (2008) Hepatic insulin resistance is sufficient to produce dyslipidemia and susceptibility to atherosclerosis. Cell Metab 7: 125-134.

2. Mori T, Kondo H, Hase T, Tokimitsu I, Murase T (2007) Dietary fish oil upregulates intestinal lipid metabolism and reduces body weight gain in C57BL/6J mice. J Nutr 137: 2629-2634.

3. Nogueiras R, Wiedmer P, Perez-Tilve D, Veyrat-Durebex C, Keogh JM, et al. (2007) The central melanocortin system directly controls peripheral lipid metabolism. J Clin Invest 117: 3475-3488.

4. Tanegashima K, Okamoto S, Nakayama Y, Taya C, Shitara H, et al. (2010) CXCL14 deficiency in mice attenuates obesity and inhibits feeding behavior in a novel environment. PLoS One 5: e10321.

5. Choi SJ, Kim F, Schwartz MW, Wisse BE (2010) Cultured hypothalamic neurons are resistant to inflammation and insulin resistance induced by saturated fatty acids. Am J Physiol Endocrinol Metab 298: E1122-1130.

6. Mysliwiec J, Zbucki R, Winnicka MM, Sawicki B, Nikolajuk A, et al. (2007) A crucial role of interleukin-6 in the pathogenesis of thyrotoxicosis-related disturbances of bone turnover in mice. Horm Metab Res 39: 884-888.

7. Wang P, Liu J, Li Y, Wu S, Luo J, et al. (2010) Peroxisome proliferator-activated receptor {delta} is an essential transcriptional regulator for mitochondrial protection and biogenesis in adult heart. Circ Res 106: 911-919.
